# Supplementary material for: Early CSF Biomarkers and Late Functional Outcomes in Spinal Cord Injury. A Pilot Study
Source: Int J Mol Sci. 2020 Nov 27;21(23):9037. doi: 10.3390/ijms21239037 (PMC7729583; doi:10.3390/ijms21239037)

Supplementary Figure 1.

Dotplots showing the individual levels of CSF biomarkers by ASIA grade.

a) Cytokines and chemokines

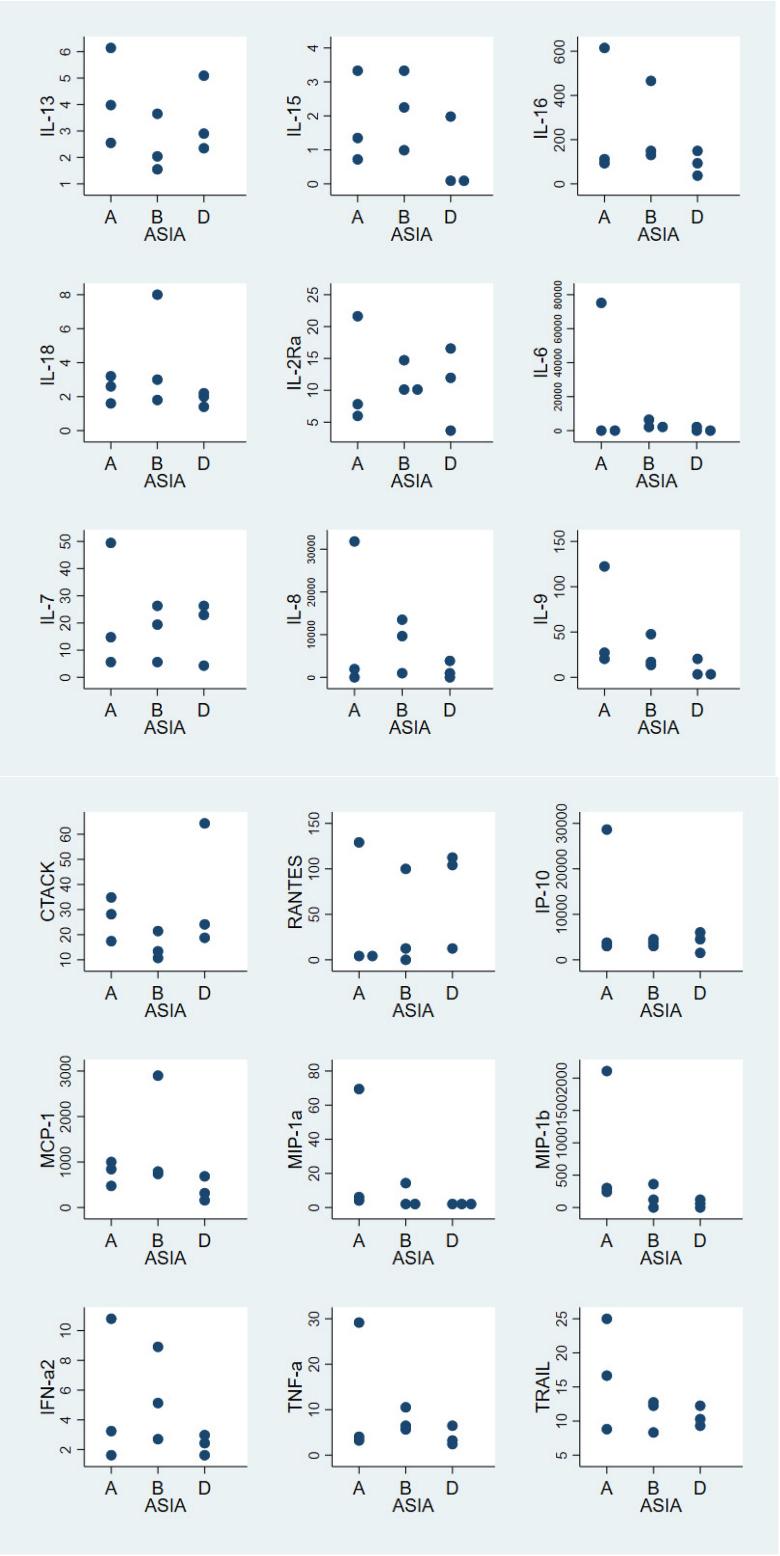

b) Growth and other factors

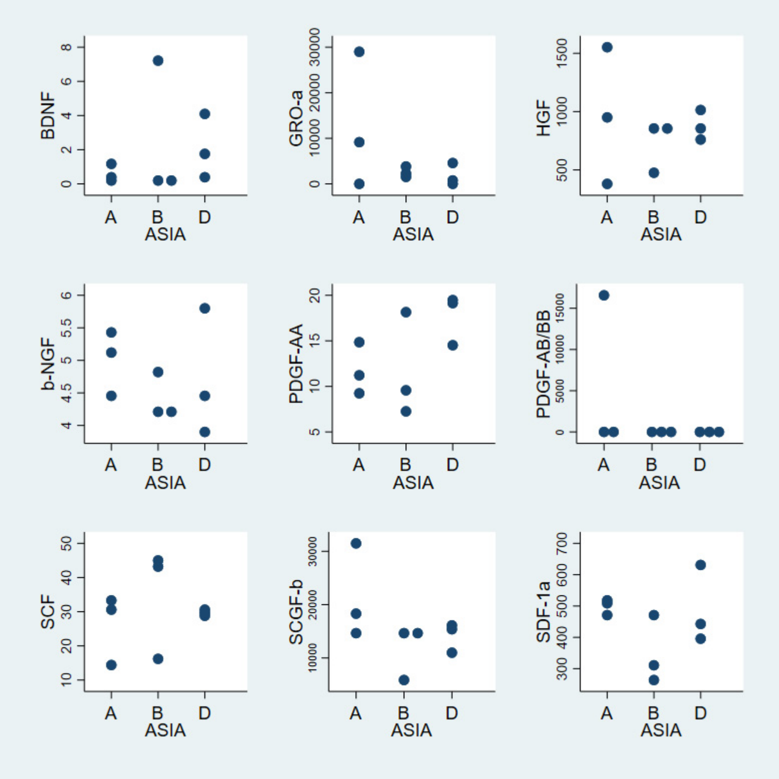

c) Soluble cell adhesion molecules and Neurological biomarkers

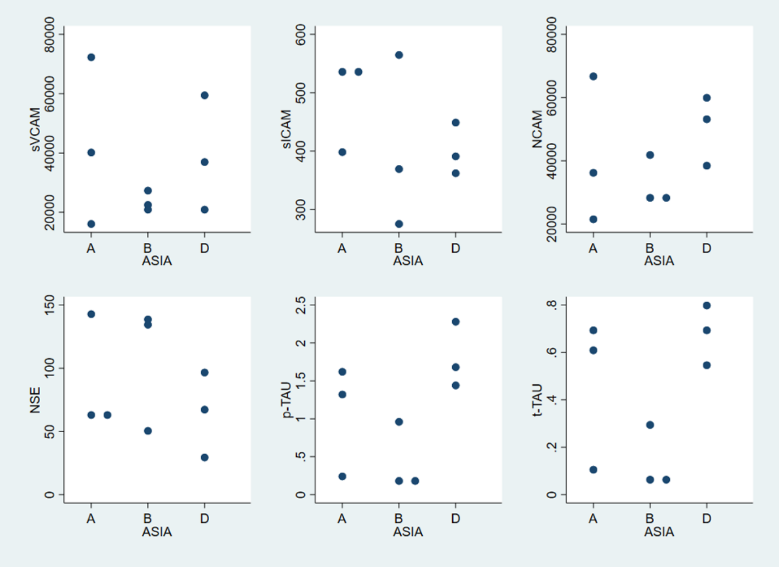

d) Other Biomarkers.

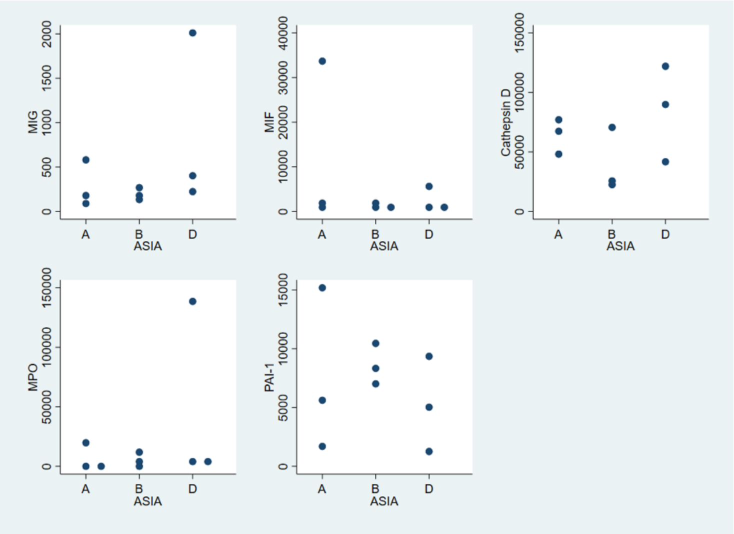

Supplement: Supplementary file 1 [file ijms-21-09037-s001.pdf]
